# A model of marmoset monkey vocal turn-taking

**DOI:** 10.1098/rspb.2024.0150

**Published:** 2024-07-03

**Authors:** Dori M. Grijseels, Daniella A. Fairbank, Cory T. Miller

**Affiliations:** ^1^ Cortical Systems and Behavior Lab, University of California San Diego, La Jolla, CA, USA; ^2^ Neurosciences Graduate Program, University of California San Diego, La Jolla, CA, USA

**Keywords:** marmosets, turn-taking, conversations, communication

## Abstract

Vocal turn-taking has been described in a diversity of species. Yet, a model that is able to capture the various processes underlying this social behaviour across species has not been developed. To this end, here we recorded a large and diverse dataset of marmoset monkey vocal behaviour in social contexts comprising one, two and three callers and developed a model to determine the keystone factors that affect the dynamics of these natural communicative interactions. Notably, marmoset turn-taking did not abide by coupled-oscillator dynamics, but rather call timing was overwhelmingly stochastic in these exchanges. Our features-based model revealed four key factors that encapsulate the majority of patterns evident in the behaviour, ranging from internal processes, such as particular states of the individual driving increased calling, to social context-driven suppression of calling. These findings indicate that marmoset vocal turn-taking is affected by a broader suite of mechanisms than previously considered and that our model provides a predictive framework with which to further explicate this natural behaviour at both the behavioural and neurobiological levels, and for direct comparisons with the analogous behaviour in other species.

## 1. Introduction

Reciprocal vocal exchanges, such as chorusing and ‘conversations’, are common across a diversity of vertebrate species, including humans [1], primates [2], mice [3] and birds [4]. These interactions are governed by a system of social rules, some of which are species specific and others shared across taxa [5,6]. A classic example of the latter is turn-taking: the behaviour of speakers alternating the timing of their successive vocalizations in a sequence. The widespread occurrence of turn-taking offers the opportunity to leverage similarities and differences in this behaviour to elucidate the underlying mechanisms of this dynamic behaviour [5]. Whereas core sensory and motor mechanisms are inherent to vocal turn-taking, speakers must also attend closely to all conspecifics in the scene and adapt their behaviour in response to ongoing contextual changes in the social and ecological landscapes. Turn-taking, after all, emphasizes the fact that conversations reflect a social interaction that is not only supported by sensory and motor processes but also a suite of complementary cognitive and state-based processes that are each necessary for if, when and how these coordinated exchanges manifest. Explicating how each of these processes complements each other is crucial to understanding the dynamic neurobiological computations that underlie natural communication behaviours and potential mechanistic differences that support seemingly similar behaviours across species.

The common marmoset (*Callithrix jacchus*), a small New World primate, engages in vocal turn-taking during antiphonal *‘*conversations’ [7–12] that is characterized by a lack of overlapping phee calls [7,10,13], as well as coordinated call timing and duration [9,10,14–16]. Recently, it was proposed that like humans [17], marmosets act as coupled oscillators such that the timing of phee call production between two marmosets will become coupled and entrained during conversations [7]. This vocal behaviour, however, is not entirely accounted for by this hypothesis, as marmosets will alter their call timing to an individual call, depending on its features [18] and do not reliably respond to the same call under similar circumstances [16]. Likewise, internal factors affect marmoset vocal behaviour in some contexts as evidenced by the fact that the distance between individuals affects their heart rate, and by extension arousal [19]. Although both internal and external factors affect marmoset vocal behaviour, how these various factors combine mathematically to drive vocal behaviour is not well understood. As such, a quantitative-driven model of marmoset conversations is needed that fully encapsulates the various factors affecting turn-taking in this primate species.

Here, we sought to characterize turn-taking during marmoset conversations by recording a large and diverse representative dataset (42 subjects of both sexes and various ages and familial status; 11 614 phee calls) and then leveraging these data to develop a novel model of natural turn-taking dynamics. We first tested and failed to replicate the coupled oscillator hypothesis for the larger population of marmosets tested here, instead finding that respective call timing between marmosets in these exchanges was stochastic. Following a thorough analysis of marmoset vocal behaviour, we propose a novel features-based model, based on internal and external states, that describes conversational dynamics across various paradigms from single, pairs and trios of marmosets, and provides a framework for the neural mechanisms underlying these dynamics [20–22].

## 2. Results

### (a) Marmoset monkeys do not act as coupled oscillators during turn-taking

To examine the dynamics of marmoset conversations, we employed a two-monkey paradigm consistent with previous studies [10,23] (electronic supplementary material, figure S1*a*; see §4 for details). A primary aim of this study was to record a large corpus of recording sessions from animals across different ages, sexes and social relatedness to accurately characterize the species’ conversational dynamics. To this end, we recorded the vocal behaviour of 42 marmoset monkeys, including 59 male–female pairs, 21 female–female pairs and 27 male–male pairs (electronic supplementary material, figure S1*b*). Twenty-five pairs (23%) were cagemates, including seven bonded pairs, 14 were parent–sibling pairs and four were siblings (electronic supplementary material, figure S1*c*). The average age of subjects at recording was 1179 days (range 260–2770 days; electronic supplementary material, figure S1*d*), and recordings were performed at varying times of the day (electronic supplementary material, figure S1*e*). While duets are limited to paired males and females in many species [24,25], conversations in marmosets occur between individuals of various ages, ranks and sexes suggesting that differences may emerge in patterns of this vocal behaviour along these different social categories. In contrast to previous studies [10,15], however, we found no effects of sex, age, cagemate status or time of day on the response rate (electronic supplementary material, figure S1*f*–*i*), possibly owing to our much larger cohort preventing confounds that may have affected previous smaller studies.

**Figure 1.**
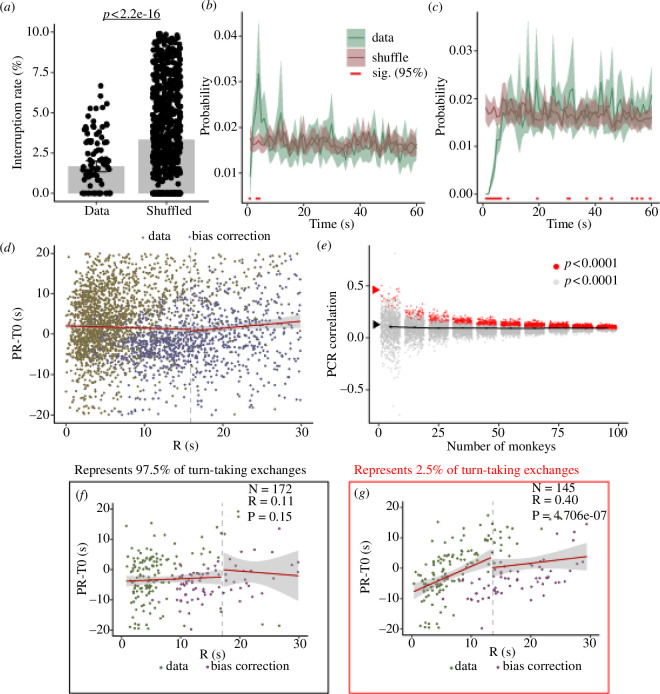
Replication of analyses showing monkeys do not act as oscillators. (*a*) Percentage of calls interrupted compared to a shuffled control. Proximate measures (replicated from Takahashi *et al*. [7]) of cross-correlation (*b*) and autocorrelation (*c*) across all sessions; data are shown in green, shuffled controls in maroon. Times where the data are significantly different from the control (Wilcoxon rank-sum test with Holm–Bonferroni correction) are indicated with a red bar at the bottom. (*d*) PRC plot of the vocal exchanges. (*e*) PRC correlation values at different subset sizes, red dots indicating datasets with a significant correlation (*p* < 0.0001). The red arrow points to dataset shown in *(f*), black arrow shows (*g*). (*f*) PRC of a representative median 10-monkey subset (*n* = 172 calls), showing a non-positive PRC correlation. (*g*) PRC of a single 10-monkey subset (*n* = 145 calls), showing a positive PRC correlation.

The current leading theory for marmoset turn-taking dynamics is that monkeys in a pair act as coupled oscillators [7]. This hypothesis states that monkeys call in an anti-phasic manner and the timing of one animal’s response determines the timing of the subsequent response in the partner. Using the dataset collected here, we applied the identical analyses [7] to test whether the coupled oscillator theory generalized to a larger more representative population of marmosets. We first confirmed that consistent with Takahashi *et al*. [7], marmosets in the current study exhibited a significant decrease in interruption rate compared to a shuffle control, indicating that marmosets actively avoid overlapping calls (figure 1*a*). While interruptions were rare in the current study, only accounting for 234 calls out of 11 450 calls (2.04%), 61.7% of our sessions had at least one overlapping call. We initially hypothesized this may be due to the inclusion of younger monkeys [26]; however, there was no significant correlation between age and the number of interruptions (electronic supplementary material, figure S1*m*).

We next tested whether our monkeys showed an oscillatory pattern in the timing of responses after a partner call by replicating the analysis performed by Takahashi *et al*. [7]. Specifically, data were analysed to determine whether the observed cross-correlations and autocorrelation showed an oscillatory pattern. In contrast to Takahashi *et al*., however, we observed neither an oscillatory pattern in our data (figure 1*b*) nor an oscillatory pattern evident in a proximate measure for the autocorrelation of the monkeys (figure 1*c*). These findings do not support the idea that marmosets act as oscillators during turn-taking, a necessary requirement for a coupled oscillator dynamic. To demonstrate that marmoset behaved as a coupled oscillator in their conversations, Takahashi *et al*. [7] showed a positive correlation between the time between two calls by the same monkey (PR), corrected for the average time between two calls by that monkey when not in conversation (T0) and the time between a monkey’s and its partner’s call (R). Yet, here we found no such correlation between R and PR-T0 (figure 1*d*). In fact, the PR-T0 in this analysis does not cross the 0 line, indicating that a long interval by monkey 1 did not result in a shorter response latency by monkey 2. As such, these findings do not support the hypothesis that turn-taking in marmoset conversations follows coupled oscillator dynamics, in stark contrast to previous work [7].

Since our results differed drastically from the previous findings, we tested whether subsets of our data consistent in size with the previous study [7] would yield more comparable results. We randomly sampled subsets of our data and performed the PRC analysis on these subsets. Results indicated that the correlation coefficient averaged around −0.01 regardless of dataset size, but there was a large range in correlations especially when only including five sessions (−0.74 to 0.81; figure 1*e*), like in the previous study [7]. This analysis revealed that the overwhelming majority of sessions (97.5%) resulted in a non-significant, or significant negative correlation (figure 1*f*), while only a few outlier subsamples (defined as any samples with *p *< 0.0001 and *r* > 0, total of 25 samples (2.5%)) from our dataset could replicate the previous effects [7] (figure 1*g*). Together, these results clearly show that although marmoset turn-taking may be consistent with a coupled oscillator dynamic on a small percentage of occasions, this mechanism is not representative of marmoset conversations.

### (b) Turn-taking probability and timing is most strongly predicted by marmoset volubility

**Figure 2.**
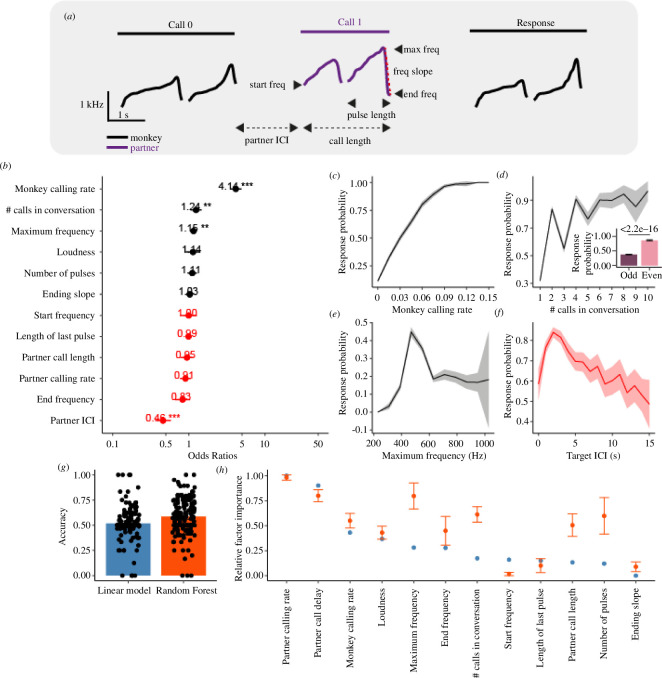
Factors affecting the response probability of marmosets. (*a*) Example call interaction showing how the various call parameters are measured. (*b*) The odds ratio of all 12 parameters as resulting from the generalized linear mixed-effects model. Effect of (*c*) monkey calling rate, (*d*) position of the call in the conversation, (*e*) maximum frequency of the call, and (*f*) the target ICI on the response probability. (*g*) Accuracy of a linear model and random forest classifier on predicting whether a call will occur. (*h*) Relative factor importance (normalized) of the linear model (blue) and random forest (orange).

To develop a model that accurately characterized the dynamics of marmoset vocal turn-taking, we first sought to thoroughly quantify the vocal behaviour and call acoustics, and to test if any of these features were predictive of whether and when a marmoset would call in a conversation. For each call, we calculated 12 parameters (figure 2*a*; see §4).

We fitted a generalized linear mixed-effects model (GLME) to the marmoset vocal behaviour data with these 12 parameters as our fixed effects [27] (marginal *R*^2^ = 0.47; see §4 for details). This showed four significant factors (figure 2*b*): the mean calling rate of the monkey (figure 2*c*), the position of the call in the conversation (figure 2*d*), the maximum frequency of the previous call (figure 2*e*) and the previous intercall interval (ICI) (figure 2*f*). Although there was a general trend of the call probability increasing with the position of the previous call in the conversation, a sawtooth pattern emerged with odd-numbered calls in the sequence exhibiting a significantly lower probability than even numbers (figure 2*d*, inset). Further analyses and modelling work discussed in upcoming sections (electronic supplementary material, figure S4*f*) suggested this was an emergent feature, and not owing to additional information present in preceding calls.

Next, we quantified features of the vocal response latency, the main factor predicted by the coupled oscillator model [7]. We combined all factors into a linear mixed-effects model, with the same fixed and random factors as above, and response delay as the dependent factor, and found that only the calling rate of the responding monkey significantly affected the response delay (electronic supplementary material, figure S2*a*). Like previous studies [14], most marmoset vocal responses recorded in the current study occurred ~3 s after the offset of the conspecific call (electronic supplementary material, figure S2*b*) and marmosets that called at a higher rate were more likely to respond to a conspecific’s call more quickly (electronic supplementary material, figure S2*c*), while the partner call delay did not affect the response delay. Consistent with our analyses above, these results further demonstrate that a coupled oscillator mechanism does not characterize marmoset vocal turn-taking and the response latency during turn-taking at the level of single calls cannot be accurately predicted with the tested factors.

These analyses indicated a relationship between the aforementioned four factors and marmoset response probability. However, this finding does not necessarily mean that these factors can predict whether an individual call within a session will receive a response by the conspecific partner. To test this issue further, we trained two classifiers: a logistic regression and a random forest classifier using the 12 factors described above, as well as an additional factor describing whether the call was an odd or even call in a conversation, on data counterbalanced per session. The logistic regression was not able to predict a response significantly above chance (figure 2*g*; *p* = 0.072, Student’s *t*‐test), suggesting no linear relation between the tested factors and the response probability, while the random forest classifier did predict significantly above chance (59% accuracy; figure 2*g*; *p* = 1.434 × 10^−7^), indicating a nonlinear effect may be at play.

When considering the predictor importance based on the out-of-bag error, the partner’s calling rate is the top predictor (figure 2*h*). This finding suggests that the volubility of each marmoset, rather than acoustic properties or vocal behaviours, is the main factor in our model determining whether or not a monkey will respond to a given call. In other words, a marmoset exhibiting high volubility in a session will be more likely to produce a response to hearing a conspecific’s call, and a marmoset will receive more responses when conversing with a partner with a high volubility. The large remaining unexplained variance in our models may be because either we did not measure all relevant behavioural characteristics (i.e. head-turning [12] and arousal [19,28]), or a stochastic process is taking place that affects marmosets’ response probability.

### (c) A novel model of marmoset turn-taking

Here, we propose a model of marmoset vocal turn-taking that combines an individual’s volubility with several internal and external modulators, which together determine a response probability. We based the modulators on both the data presented here, as well as existing studies in the relevant literature, including previous studies showing the effect of various internal [19,20,28] and external [18,29] factors affecting marmoset calling, and relevant modelling work [18,30]. Although our model uses a similar general combination of factors, we explicate these factors in more detail, show how the observed and modelled behaviour arises from modelled neuronal activity, and explain how they could relate to known neural correlates. Together this provides a more comprehensive features-based model that illustrates the complexity of vocal turn-taking and the myriad complementary factors that influence its occurrence.

**Figure 3.**
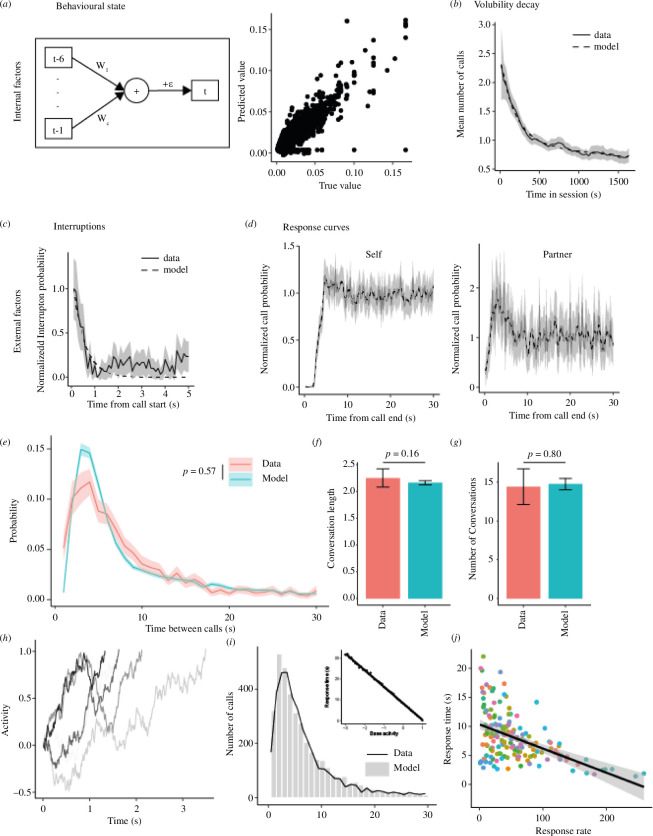
Overview of novel marmoset phee communication model. The first row represents the internal factors (*a*) internal state, as modelled by a linear model with the structure illustrated left, which can predict the state value based on the preceding five timepoints (right). (*b*) Arousal, as modelled by an exponential decay across the session (dashed). External factors include (*c*) interruption curve, as modelled by an exponential decay (dashed), and (*d*) response curves, obtained from the data. (*e*) The intercall interval of the data (pink) compared to the model (blue). The mean conversation length (*f*) and number of conversations (*g*) of the data (pink) compared to the model (blue). (*h*) Example of 5 epochs of ramping activity, leading to varying response delays. (*i*) Population response delay as modelled using the ramping model (grey bars), and measured from the data (black line). The inset shows the relationship between the base activity and the mean response delay. (*j*) Measured relationship between the response rate in a single session and the response time. Each dot shows a single monkey in a single session.

Most marmosets exhibited variability in their calling rate across recording sessions (see, for example, electronic supplementary material, figure S3*a*), likely reflecting changes in arousal [28], behavioural context [18,20] and other internal processes, which we jointly label ‘behavioural state’. We postulate this factor may reflect spontaneous fluctuations of the resting state [31], which causes changes in excitability over time, and consequently changes in volubility. The covariation in these behavioural state fluctuations between each pair of monkeys did not significantly differ from shuffled controls (electronic supplementary material, figure S3*b*). A linear model was used to produce novel session-wide behavioural state fluctuations as inputs (figure 3*a*; see §4 for details). In addition to independent state-wide fluctuations, the overall trend in the monkeys’ calling rate decreasing over time was labelled as ‘volubility decay’ (figure 3*b*). We believe this may reflect a combination of arousal, interest, motivation and unknown other factors that are difficult to determine from the features measured here. Given the current data, however, the most accurate representation was to fit an exponential decay function (figure 3*b*, dashed line) to model the decreasing volubility of the monkey over the span of a session.

Turn-taking is affected by at least two key external factors related to the vocal behaviour of the other monkey in the conversation. First, marmosets largely avoid interrupting conspecific calls [7,10,26] (figure 1*a*). Analysis of the timing of interruptions in the current dataset found that the majority of calls were interrupted within the first 0.5 s (figure 3*c*), likely representing covocalizations, where both marmosets initiate a call at approximately the same time, with a rapid decline over time. This was modelled using an exponential decay, representing the rapid decline in the likelihood of calling by a monkey while the partner is vocalizing [17,32–34]. Second, we analysed the average call probability of the marmoset producing a call immediately following the offset of its previous call and revealed a refractory period of about 2.2 s (figure 3*d*). While a small portion of this duration is the result of physical constraints, i.e. the monkey needs to take in a breath before producing a subsequent phee, much of this period is likely active suppression of vocal production to allow for the other conspecific turn to respond. Following this refractory period, a rapid rise in call probability occurred before returning to baseline around 4.6 s after the offset of the previous call. The partner marmoset exhibits a complementary pattern of vocal behaviour. Both these factors represent volitional control of vocalizations, with likely neural correlates in the prefrontal and premotor cortex [35].

Our novel turn-taking model combines the four factors described above—behavioural state, volubility decay, interruptions, and response curves—to represent the calling probability as described in §4. We were able to simulate marmoset conversations and generated ‘turn-taking’ using a fivefold cross-validation paradigm. To validate the model from the test set, the calling rate of our actual sessions was input into the model and the resulting dataset analysed (see figure 3*f*, e.g. interactions from the data and model). The resulting ICI probability density distribution, a measure used previously to determine model accuracy [7], was not significantly different from the data ICI (figure 3*g*). Moreover, no significant differences were evident between the model and the data for the number and length of the conversations emerging from the models (figure 3*h,i*). To test the individual effects of the four factors—behavioural state, volubility decay, interruptions and response curves—each was excluded separately and the changes to the conversation dynamics were quantified. Analyses revealed that only the response factor significantly affected the ICI distribution (electronic supplementary material, figure S4*a*) and the length (electronic supplementary material, figure S4*b*) and number (electronic supplementary material, figure S4*c*) of the conversations. Finally, the same GLME was applied to the modelled data, excluding the call length and frequency factors as these were not varied in the model, which showed significant effects of monkey call rate, partner ICI and conversation number (electronic supplementary material, figure S4*d*–*h*). In addition, the sawtooth pattern that was observed in the effect of the position of the call in a conversation on the response probability was evident in the modelled data (electronic supplementary material, figure S4*f*). Neither the partner ICI nor the conversation number effects were explicitly modelled, suggesting these results in the data were emergent from the conversation structure and calling rate, rather than causative results.

Overall, our model of marmoset turn-taking accurately captures the conversation dynamics of dyads. It is, however, important to consider that although the other factors did not significantly affect the ICI or conversations in this social context, they do reflect true dynamics of the behaviour (e.g. a lack of interruptions) and may be more integral in more complex social contexts.

### (d) Vocal behaviour across different social contexts

The vocal turn-taking model shown above effectively captures the dynamics of marmoset dyads in a conversation. However, this is not the only context in which marmosets engage in conversational turn-taking. While single marmosets may sometimes be isolated from the group, more commonly multiple marmosets are present in a scene and converse in dynamic communication networks. To test whether vocal behaviour generalizes to other behavioural contexts, and how well our turn-taking model performs in these conditions, we tested single animals and trios of visually occluded marmosets.

**Figure 4.**
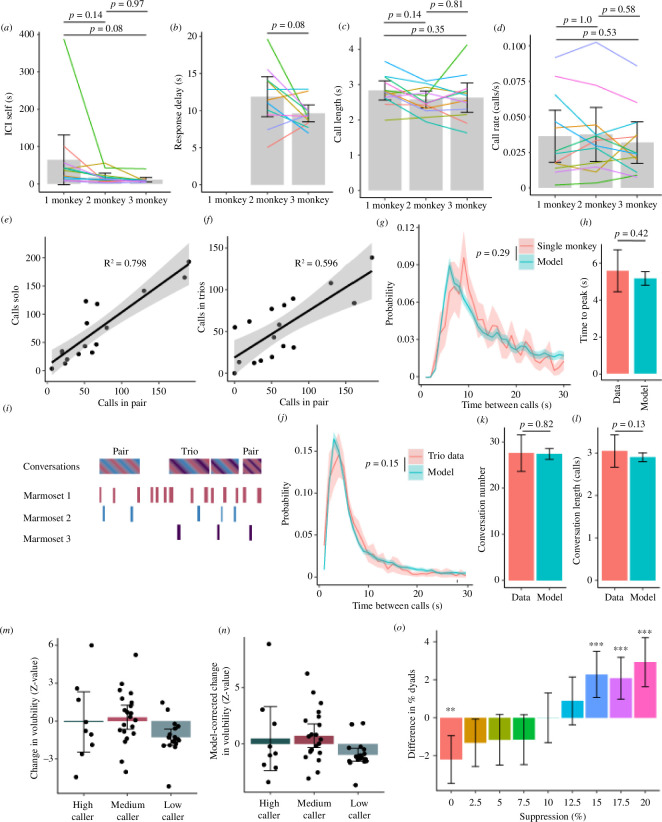
Novel marmoset communication model applied to novel paradigms. (*a*) ICI between consecutive calls by the same monkey across the three paradigms. (*b*) Response delay between calls of monkeys engaging in conversation. (*c*) Mean call length of monkeys across the paradigms. (*d*) Calling rate of monkeys across three paradigms. (*e*) The mean calling rate of individual monkeys in the paired condition compared to the solo condition. (*f*) The mean calling rate of individual monkeys in the paired condition compared to the three-monkey condition. (*g*) The intercall interval in the data (pink) compared to the model (blue). (*h*) The mean time of peak ICI in the distribution. (*i*) Example conversation showing paired and trio conversations. (*j*) The intercall interval in the data (pink) compared to the base model. The number (*k*) and mean length (*l*) of the conversations. The (*m*) change in volubility and (*n*) model-corrected change in the volubility of the high, medium and low caller in each three-caller session. (*o*) Difference in the proportion of all conversations that included only two out of the three monkeys. *p < 0.05, **p < 0.01, ***p < 0.001, ****p < 0.0001.

First, we compared the vocal behaviour of the 12 subjects tested in all three test contexts: solo, paired and trios. Although trends towards shorter and faster calls, when more monkeys were interacting, were present across the data, no statistical differences in the mean ICI between subsequent calls by a monkey (ICI self; figure 4*a*), the response delay (figure 4*b*) or call length (figure 4*c*) were evident. Marmosets also did not change their calling rate across contexts (figure 4*d*), and the calling rates in both the solo (figure 4*e*) and trio (figure 4*f*) conditions were highly correlated to the paired calling rate for individual monkeys, suggesting that individual differences in volubility were stable across these social contexts.

Our model based on paired monkeys was then applied to test whether it could accurately predict marmoset vocal behaviour in the solo context. This resulted in an ICI distribution that was not significantly different from the two-monkey experiment (*p* = 0.29, linear mixed-effects model; figure 4*g*), suggesting our model broadly captured the vocal behaviour of single marmosets. However, a (non-significant) delay in peak ICI in the behaviour data compared to the model was evident (figure 4*h*; *p* = 0.42). Based on the trends in the two-monkey data, we conjectured that this may reflect an interruption avoidance strategy of the marmosets. Specifically, marmosets reduce their response delay and call length when other marmosets are present in the scene, which is reflected in the shorter peak ICI in the modelled data. To test whether our model generalized to more complex social scenes, we examined marmoset vocal behaviour in a social context comprising three visually isolated marmosets (see §4). A sample of call interactions in these three monkey sessions is shown in figure 4*i*. The ICI distribution in this multi-monkey context was not significantly different from that in the two-monkey contexts (*p* = 0.15, linear mixed-effects model; figure 4*j*). Neither the number of conversations (figure 4*k*) nor the length of the conversations (figure 4*l*), was significantly different from the model conversations.

Overall, these results indicate that the structure of marmoset turn-taking is largely consistent in multi-caller scenes, despite vocal interactions being modulated by social context in other species [12,36,37]. It is, however, important to note that the model is based on the measured volubility and temporal dynamics of conversations during each session, so individual differences in behaviour and volubility, whereby certain monkeys are suppressed while others increase their volubility would not be captured by these initial analyses. Indeed, measuring the relative change in the volubility of monkeys compared to their previously measured volubility in the solo and paired experiments revealed that on average one caller in each session significantly down-modulated their volubility (figure 4*m*). This effect remained in the model-corrected volubility (see §4), which corrects for the effects of the other monkeys’ measured volubility (figure 4*n*). This suggests that a social suppression factor should be included in the model to account for scenes comprising multiple marmosets. We modelled a suppression factor for the non-conversation partner during ongoing conversations with the other individuals by testing nine levels of suppression (0–20% with 2.5% steps). Comparisons of these suppression models to the data showed that although no model captures all aspects, the 10% model shows the most similar proportion of dyads to the data and the closest fit with the data overall (figure 4*o*; electronic supplementary material, figure S5*a*–*d*). This suppression model exhibited an improvement over the base model, as the conversations it produces are more similar in structure to the data, suggesting a conversation-specific socially mediated reduction in volubility is present in multicaller groups. What remains unclear, however, is what determines which individuals suppress their calling at the outset of a session (i.e. low caller) and why the conversationalists change when they do. This effect highlights the fact that key sources of variance in this behaviour have yet to be incorporated into our model and that targeted experimental work is likely needed to fully account for certain facets of marmoset vocal turn-taking.

## 3. Discussion

Here, we propose a new features-based model of marmoset vocal turn taking. Leveraging the extensive analyses of marmoset vocal behaviour in multiple behavioural contexts described here, we show that marmoset vocal turn-taking during conversations involves a suite of complementary processes that collectively**—**and respectively—influence this behaviour that had not collectively been considered previously. We did not find evidence that the marmosets act as coupled oscillators, and suggest that previous evidence for this was caused by a small sample size—potentially resulting in the inadvertently biased population (i.e. highly ‘vocal’ monkeys might be more likely to be selected to be experimental animals)—that does not generalize to a larger and more representative population. Although previous studies found varying effects of social and behavioural factors [28,30,38,39], these had not previously been combined in a comprehensive model of marmoset vocal interactions. Our model of marmoset turn-taking revealed that at least four separable factors have differential effects on vocal behaviour. Importantly, the independent influence of these factors was not solely evident from behavioural analysis, but only became apparent when modelling each source of potential variance affecting marmoset conversational dynamics in different contexts. More complex multi-speaker environments revealed that other social factors may affect these vocal interactions. Conversations, for example, bias to dyads and the conspecific in the scene who is not in a conversation will suppress their calling during the interaction. Our turn-taking model illustrates that multiple distinct behavioural factors underlie the dynamics of vocal turn-taking across social contexts and provides a new framework for explicating its underlying mechanisms.

The four factors affecting marmoset vocal turn-taking revealed by our model provide a key foundation upon which to further elucidate this dynamic behaviour in marmosets and other vertebrate species, at the behavioural and neural levels. Like marmosets, meerkats exhibit interruption avoidance and increased call probability after hearing a conspecific call [34]. Both singing mice [33] and naked mole rats [40] similarly show response onset linked to conspecific calls, though at significantly faster intervals than is observed in marmosets. Zebra finches show both a refractory period after a self call, and an increased call probability after hearing a conspecific call [41]. Other species, such as elephants [37], alter their interruption rate based on the conversation partner. We conjecture that our model can serve as a framework to identify shared and unique processes underlying turn-taking across taxa. The similarities and differences across species in our model would potentially make key predictions about the underlying neural mechanisms and by extension a path for elucidating the evolution of the neural circuits underlying coordinated vocal interactions (i.e. turn-taking).

One notable limitation of our study is that we could not account for all sources of variance in the vocal behaviour, a feat that has not yet been achieved in these types of studies. While some elements of marmoset conversations appear stochastic, they may simply not yet be accounted for by the model. The addition of further behavioural quantification, such as body posture, overall arousal and more detailed analysis of state fluctuations can further refine the model and increase its explanatory power. Indeed, a previous report [28] suggested arousal to be an important driver of individual vocalizations, but its role in antiphonal settings has not yet been explored. This would also increase the generalizability of the model, as in the current data, the monkeys are brought into an artificial environment, and cannot physically move towards each other. However, in a wild setting, phee calls were often followed by a movement of conspecifics to the sound [12]. As occurred following analysis of the multi-caller contexts, the model can be updated with additional factors to account for such discrepancies and describe the underlying dynamics in more detail. In addition, as our model is stochastic in nature, it has limited predictive power for the timing of individual calls in conversations, but rather simulates new conversations that match the data in overall structure. Although the probability calculated by the model may be used to predict responses over short time periods, it does not predict session-wide conversations. Finally, although our model accurately describes the contexts tested here, it does not yet account for both the rapid [13,42] and structural [9] adaptations the monkeys are able to make in the presence of interfering noise. This behaviour is likely crucial to their vocal communication, as it is needed to effectively communicate in an environment with competing background noise [35].

The factors included in our vocal turn-taking model are based on analysis of the behavioural phenotype, but they represent the underlying neural mechanisms or suites of mechanisms that support marmoset conversations. The model is based on a probability measure, calculated using the base probability and the various aforementioned factors. We proposed that this probability-based approximation may manifest as neural activity with random variability crossing a certain threshold to initiate a call, reminiscent of decision-making dynamics [18,43,44], and showed that such neural activity could explain the observed behaviour. Several prefrontal cortex regions have been indicated in decision-making studies [45], as well as the anterior cingulate cortex [45–47]. Given its role in vocal control [48], this area is a promising candidate for further studies into the proposed ramping model of phee responses during marmoset conversation. Our model further includes a behavioural state which fluctuates independently in each animal, suggesting a lack of cross-session coordination between animals [45,46]. The turn-taking model described here provides a powerful framework to better understand the processes affecting this natural vocal behaviour and a roadmap for explicating the neural mechanisms that underlie each of these factors, both in these marmosets and more broadly for comparative analysis of other species. However, it should be noted that while turn-taking is a core feature of ‘conversations’ in many vertebrate species, it is not entirely equivalent to human turn-taking. As such, there may be limitations when applying our model to human vocal behaviour.

## 4. Methods

### (a) Subjects

A total of 53 unique subadult and adult marmosets (*C. jacchus*) were used across the three experiments. For the two monkey experiments, a total of 42 unique monkeys were included (48% female), with ages between 284 and 2737 days at the time of recording. For the single-monkey experiments, 27 unique monkeys were included, of which 17 were also included in the two-monkey recordings (59% female), with ages between 331 and 3421 days at the time of recording. For the three-monkey experiments, 21 unique monkeys were included, of which 19 were also included in the two-monkey recordings (48% female), with ages between 572 and 2466 days at the time of recording. Twelve monkeys were tested in all three behavioural contexts. All experiments were approved by the Institutional Animal Care and Use Committee.

### (b) Experimental setup

All recording sessions took place in a radio-frequency shielding room (ETS-Lindgren) in a 4 × 3 × 3 m^3^ room. Animals were placed in a 32 × 18 × 46 cm^3^ box, with a mesh on one side, and placed on a table on either side of the room. For the three-monkey experiments, a third table was added such that the three tables formed a triangle. All boxes were separated by an opaque black curtain. Each box had a directional microphone pointed towards it (Sennheiser model MKE 600 and ME-66), which was amplified using a preamplifier (PreSonus BlueTube DP v2). Data from all microphones were acquired using a custom MATLAB script.

### (c) Coupled oscillator analyses

First, we calculated the interruption rate by determining the number of overlapping calls. We obtained the shuffled dataset by randomly combining two monkeys from different sessions and calculating the interruption rate for these shuffles. The phase response curve (PRC) was calculated as previously described [7]; in short, we calculated the response interval (R, mean time between a call and a response), phase response (PR, mean time between two calls of the same monkey, when partner called in between) and median call interval (T0, mean time between two calls of the same monkey with no calls in between) for each session. We included the bias correction points, which are any calls where PR < R. We calculated the mean T0 across all sessions and then determined the PRC correlation both before and after T0. Next, we randomly selected subsets of sessions of various sizes and performed the same PRC analyses on these subsets, to determine the effect of the number of datasets included on the PRC correlation. We calculated the PRC and *p*-value using the *corrcoef* function in MATLAB.

### (d) Predictive models

We first extracted 12 predictive factors for each call (figure 2*a*), most representing characteristics of the preceding call, and inspired by previous work [23,49]. The preprocessing and extraction were performed using a custom script (https://doi.org/10.5061/dryad.9ghx3ffpx) [50]. In short, each call was first extracted using an automated script, and then hand-corrected to obtain start and end times. The power spectrum of the identified calls was calculated with the MATLAB *pspectrum* function, and the fundamental frequency over time was extracted by taking the frequency with the maximum power in the spectrum at each timepoint, which was used to calculate all frequency-based features (figure 2*a*).

— *Monkey calling rate*: The mean cumulative calling rate of the monkey producing the call.
— *Call # in conversation*: Number of consecutive calls so far in the conversation.
— *Maximum frequency*: The maximum frequency of the call.
— *Loudness*: The relative loudness of the call, normalized to the mean loudness of all calls produced by the same monkey in that session.
— *Number of pulses*: The number of phee pulses of which the call consisted.
— *Ending slope*: The slope from peak frequency to phee offset (Hz s^−1^) of the call.
— *Start frequency*: The frequency at phee onset of the call.
— *Length of the last pulse*: The length (in s) of the last phee pulse of the call.
— *Partner call length*: The total length (in s) of the call.
— *Partner calling rate*: The mean cumulative calling rate of the partner monkey.
— *End frequency*: The frequency at phee offset of the call.
— *Partner ICI*: The time between the last call of the partner monkey and the current call.

We fitted generalized linear mixed-effects models using the *glmer* function in R [27], with the 12 factors as the fixed effects, and the monkey and partner ID as the random effects, to obtain the odds ratio. Prior to fitting the linear model and random forest, we counterbalanced the data by including an equal number of responses and non-responses per session. We fitted a linear model (function *fitclinear*) on this data to predict whether the partner would respond to this call, and calculated the prediction error to determine the accuracy. The random forest was fitted using the *TreeBagger* function in MATLAB, with the predictor selection set to ‘interaction-curvature’, and accuracy was again obtained by calculating the prediction error. We extracted the predictor importance in the linear model from the absolute weights of the predictors, and from the random forest using the built-in *OOBPredictorImportance*.

### (e) Marmoset phee communication model

#### (i) Behavioural state model

To approximate the continuous behavioural state of the animals (electronic supplementary material, figure S3*a*), we first calculated the rolling average calling rate in fifty-five 180 s windows with a step size of 30 s. We used five consecutive windows as the predictor with the subsequent window as the dependent variable in our model. We trained a linear model using the built-in *fitlm* function in MATLAB. We extracted the prediction error of the model and fitted a Gaussian distribution using the *fitdist* function. Next, to model new behavioural state values, we randomly selected five values from a Gaussian distribution fit on the calculated mean calling rates. We then recursively predicted the subsequent value using the linear model, and added a random error to this value drawn from the Gaussian distribution fit on the prediction error. We threw out the first 70 values, to prevent any effect from the initial 5 seeding values, and divided the remaining behavioural state values into 200 sessions of 70 values, each normalized to have a mean of 1. For each modelled monkey, a behavioural state vector was randomly drawn, and the vector was interpolated (*interp1* function, using *pchip* interpolation) to obtain a behavioural state value per second. To prevent edge effects from the interpolation and correlations caused by the continuous nature of the initial generation of the behavioural state, the interpolated values consistent with the values outside of the 1800 s session length were dropped. Importantly, this model does not apply to ICI.

#### (ii) Arousal

We used the same rolling average calculated for the behavioural state to obtain the mean calling rate across each session. These were averaged across all sessions and monkeys to obtain a single vector, which was normalized to have a mean of 1. We fitted this average with a two-term exponential decay model interpolated (*fit* function, using *exp2* fittype), and used the resulting model to generate the arousal decay over time for each session.

#### (iii) Interruptions

The interruption curve was calculated by determining the mean number of calls by the partner monkey while a monkey was vocalizing, calculated in 0.2 s windows for a total of 5 s. This mean number was normalized to 0–1 range, and fit with an exponential decay with an origin of 1, using the MATLAB *fit* function.

#### (iv) Response curve

We calculated the mean response curve by determining the mean number of calls by the caller and by the partner occurring in each 1 s time bin across the 30 s following the end of a call. This response curve was then smoothed for each monkey and session using the *smooth* function with a local regression using weighted linear least squares and a second-degree polynomial model. The resulting smoothed response curves were then normalized to the base rate, defined as the response likelihood in the last 10 s of the 30 s curve, and averaged to obtain the response profile.

#### (v) Correction factor

As the model varied the probability of calling depending on the behavioural state and occurrence of calls, a discrepancy in the inputted calling rate and the resulting modelled rate occurred. The size of this discrepancy depended on the inputted calling rates, and as such, we modelled a multiplicative correction factor to account for this. We first built the model as specified, and then generated model conversations for input calling rates of 0–300 calls in 10 call steps for each monkey, and calculated the discrepancy. We then fitted a linear model (*fitlm* function) to predict the discrepancy in calling rate based on the monkey and the partner calling rate. We used this correction model to correct the calling rates prior to running the model, so it generated the same number of calls as the inputted values for each monkey.

#### (vi) Model-corrected calling rate

Using the linear model for the correction factor, we can calculate the original input calling rate required to produce the output calling rates observed. As the correction is differentially affected by both the caller’s and its partner’s rate, we calculate the correction for both separately (using the same linear model) to obtain the model-corrected calling rate.

#### (vii) Conversation modelling

Each 1800 s session was divided into 0.2 s bins, and we determined a baseline probability for each bin by multiplying the calling rate of each monkey, the correction factor calculated for each monkey, the arousal curve and the behavioural state curves randomly selected for each monkey. Next, we iterated through each timepoint (i.e. 1800 × 5 timepoint total), and drew a random number for each monkey (MATLAB *rand* function). If this number was smaller than the probability of calling at that timepoint, a call occurred for that monkey. The call length was randomly drawn from a Gaussian distribution fitted to the actual call length. The probability for the subsequent time bins was altered by multiplying the appropriate interruption curves and response curves with the existing probability. We performed this action iteratively until the end of the session was reached, and outputted the list of calls and who made each call for further analysis.

#### (viii) Cross-validation

As the model was based on the two-monkey data, we applied cross-validation to generate the two-monkey datasets. The total number of datasets (*n* = 107) was divided fivefold. We then fitted the four factors on all but onefold and used the resulting factors and curves to generate the model data for fivefold. As such, the modelled session was never included in the model data.

## Data Availability

The data used to generate the figures, analyses and model are available on Dryad [50]. Supplementary material is available online [51].
